# Idiopathic eosinophilia complicated with cryptococcal meningitis: a case report

**DOI:** 10.1177/03000605241305437

**Published:** 2024-12-20

**Authors:** Zhongshuo Liu, Ying Lou, Yingxiao Ji, Congying Zhao, Litao Li

**Affiliations:** 1 Department of Neurology, 117872 Hebei General Hospital, Shijiazhuang, Hebei, China; 2 Hebei Provincial Key Laboratory of Cerebral Networks and Cognitive Disorders, Shijiazhuang, Hebei, China; 3 Hebei Medical University, Shijiazhuang, Hebei, China

**Keywords:** Idiopathic eosinophilia, cryptococcal meningitis, nervous system infection, glucocorticoid, headache, case report

## Abstract

Idiopathic eosinophilia, characterized by unexplained peripheral blood eosinophilia after ruling out secondary causes, is an extremely rare condition. Cryptococcal meningitis is a life-threatening opportunistic infection that primarily affects immunocompromised individuals, such as those with advanced AIDS or leukemia. In this report, we present a unique case of idiopathic eosinophilia concurrent with cryptococcal meningitis, which, to the best of our knowledge, is the first such case described globally.

## Introduction

Eosinophilia, defined as an abnormally high count of eosinophils in the peripheral blood exceeding 1.5 × 10^9^/L, is considered idiopathic (also known as hypereosinophilic syndrome [HES]) when secondary causes are excluded. A comprehensive diagnostic approach for eosinophilia involves various tests, including morphological examination of blood and bone marrow, standard cytogenetics, fluorescence in situ hybridization, molecular assays, and flow immunophenotyping. These tests are essential for detecting histopathological or clonal evidence of acute or chronic hemolymphoma.^1–3^

Cryptococcus is a naturally occurring yeast primarily comprising the pathogenic strains *Cryptococcus neoformans* and *Cryptococcus gatti*.^4^ These fungi are opportunistic pathogens that predominantly affect immunocompromised individuals, causing life-threatening conditions such as meningitis and, less commonly, pneumonia. Cryptococcal infections are relatively rare, with an average patient age of 45 years and a male-to-female ratio of 3.1:1.^5^ High-risk groups include individuals with HIV, those undergoing cancer chemotherapy, patients receiving high-dose corticosteroids, solid organ transplant recipients, and other individuals with compromised immune systems. The mortality rate for cryptococcal meningitis ranges from 30% to 60%, with more than 181,000 deaths reported globally each year. Without treatment, the mortality rate can reach 100%.^6^

In this report, we describe a unique case of idiopathic eosinophilia complicated by cryptococcal meningitis. To the best of our knowledge, this is the first such case reported globally.

## Case presentation

**Figure 1.**
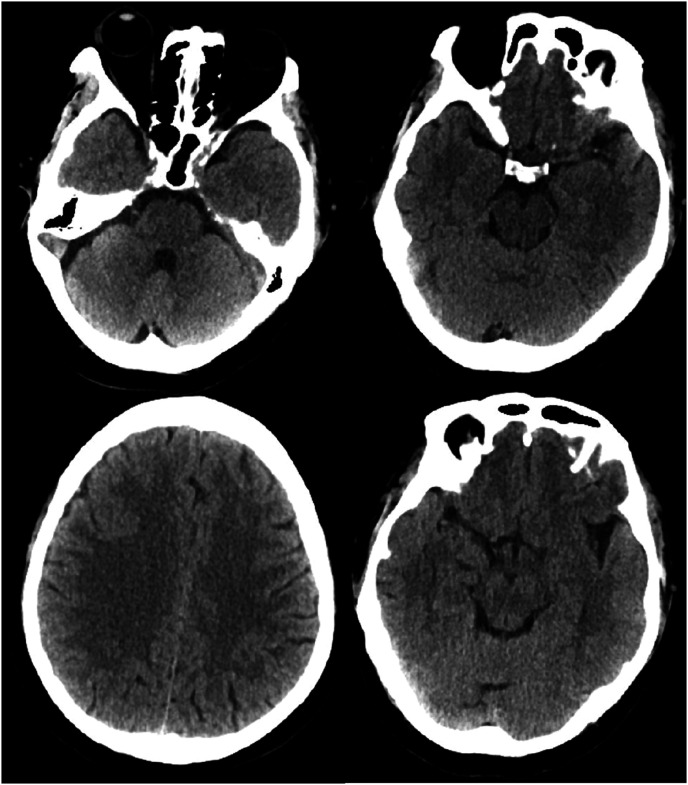
Emergency cranial computed tomography scan showing no evidence of bleeding.

**Figure 2.**
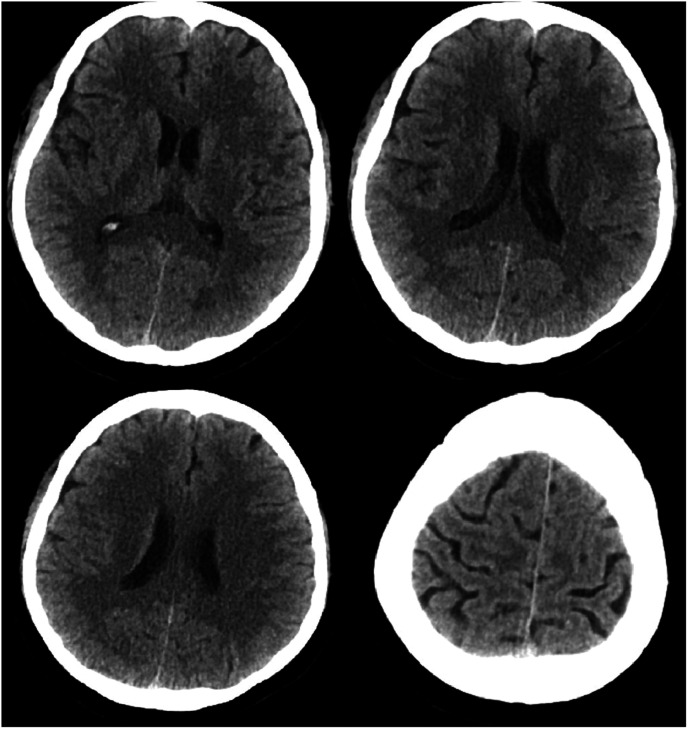
Emergency cranial computed tomography scan showing no evidence of bleeding.

A man in his early 50s presented with a 1-month history of headaches, initially centered between his eyebrows and without an apparent cause. The pain was mild and not considered serious. Two weeks before his visit, he developed bilateral posterior neck pain, which he tolerated. Five days prior to admission, while at work, the headache intensified and was accompanied by posterior neck discomfort and dizziness upon standing. He denied symptoms such as diplopia, visual disturbances, hearing loss, tinnitus, palpitations, chest tightness, or chest pain. Additionally, he had no signs of limb weakness, numbness, urinary or bowel disturbances, or loss of consciousness. One day before admission, the headache became unbearable and was accompanied by nausea, vomiting after meals, and profuse sweating. These symptoms prompted him to seek emergency care. A cranial computed tomography scan showed no evidence of bleeding (Figures 1 and 2). He was subsequently admitted to the neurology department with a preliminary diagnosis of headache of unknown origin.

The patient had a history of hypertension for more than 2 years, with blood pressures reaching as high as 200/100 mmHg. He had been diagnosed with idiopathic eosinophilia more than 2 years previously and was hospitalized multiple times in the hematology department. One year previously, he began experiencing intermittent chest tightness and shortness of breath, which persisted for more than 1 year and was occasionally accompanied by chest pain. During one hospitalization, he was diagnosed with idiopathic hypereosinophilia syndrome, coronary atherosclerotic heart disease with acute extensive anterior myocardial infarction (Killip grade I), ischemic nephropathy, chronic kidney disease (stage 2), grade 3 hypertension (very high-risk), fatty liver, pulmonary nodules, atrophy of the right kidney, right kidney stones, chronic prostatitis, hypoproteinemia, and hypocalcemia.

At discharge, he was prescribed the following medications: prednisone tablets (20 mg/day), omeprazole enteric-coated capsules (1 capsule/day), calcium carbonate tablets (1 tablet/day), perindopril tert-butylamine tablets (4 mg/day), metoprolol tartrate (12.5 mg twice/day), trimetazidine tablets (20 mg twice/day), atorvastatin calcium tablets (20 mg/day), clopidogrel (75 mg/day), aspirin enteric-coated tablets (75 mg/day), spironolactone tablets (20 mg at night), and misemide tablets (20 mg/day). These medications generally maintained his blood pressure at approximately 120/70 mmHg.

The patient also had a history of renal artery thrombosis, fatty liver, and pulmonary nodules, first identified more than 2 years previously. He denied infectious diseases such as hepatitis, tuberculosis, typhoid, or malaria. He also reported no history of diabetes, trauma, surgery, or blood transfusion. His vaccination history was unknown, and he had no reported food or drug allergies.

On physical examination, the patient was alert and cooperative. He exhibited nuchal rigidity and positive meningeal signs, while the remainder of the examination was unremarkable. There was no family history of similar conditions.

Admission auxiliary tests revealed the following. Blood analysis revealed a white blood cell count of 20.37 × 10^9^/L, with neutrophils at 67.30% (13.72 × 10^9^/L), lymphocytes at 4.00% (0.81 × 10^9^/L), eosinophils at 25.10% (5.11 × 10^9^/L), monocytes at 0.61 × 10^9^/L, and basophils at 0.12 × 10^9^/L. The C-reactive protein level was elevated at 42.78 mg/L. Emergency biochemical tests showed a total protein level of 59.98 g/L, albumin (bromocresol green method) of 38.98 g/L, sodium of 135 mmol/L, urea of 8.72 mmol/L, and blood glucose of 8.78 mmol/L. Coagulation tests showed a fibrinogen content of 4.6 g/L; the other parameters were within normal limits. Cardiac enzyme analysis showed an aspartate aminotransferase level of 14.8 U/L, creatine kinase of 39.0 U/L, and oxybutyrate deaminase of 187.2 U/L, with no abnormalities detected in troponin levels (troponin T: <40 ng/L; creatine kinase-MB (CK-MB): 12.4 U/L). An electrocardiogram revealed sinus rhythm with ST-segment elevation in leads V3–V5, T-wave inversion, and pathological Q waves in leads II, III, aVF, and V1–V5. Coagulation tests showed an activated partial thromboplastin time of 28.3 seconds, fibrinogen content of 4.6 g/L, and D-dimer quantification of 0.35 mg/L FBU. Cardiac ultrasound findings included decreased movement of the anterior wall of the left ventricle and apex, suggestive of apical ventricular aneurysm formation. Additional findings included left ventricular dilation, moderate aortic and tricuspid valve regurgitation, pulmonary hypertension, a small pericardial effusion, and grade II left ventricular diastolic dysfunction.

The patient’s headache persisted after admission. There were no significant abnormalities in eight preoperative tests, including troponin T measurement, tumor marker measurement, thyroid function tests, and urinalysis with urine sediment examination. Biochemical analysis revealed the following results: total protein, 54.9 g/L; albumin, 34.8 g/L; lactate dehydrogenase, 332.2 U/L; CK-MB, 19.9 U/L; sodium, 132 mmol/L; chloride, 98 mmol/L; uric acid, 151.4 µmol/L; triglycerides, 5.22 mmol/L; high-density lipoprotein, 1.67 mmol/L; and N-terminal B-type natriuretic peptide precursor, 4652 pg/mL. Cervical vascular ultrasound showed bilateral common carotid artery plaque formation with a stenosis rate of <50% and reduced flow velocity in the bilateral internal carotid arteries. Plaque formation was also observed in the right subclavian artery, with a stenosis rate of <50%. Ultrasound of the lower limbs revealed no abnormalities in the deep veins or interscalene region. However, deep artery ultrasound indicated occlusion of the right posterior tibial artery.

After admission, the patient experienced severe headaches, bilateral posterior neck pain with local tenderness, nausea, and gastric, non-projectile vomiting. Routine management included analgesia, dehydration therapy, and measures to reduce intracranial pressure. A lumbar puncture was performed, revealing an opening pressure exceeding 330 mmH_2_O. Approximately 20 mL of cerebrospinal fluid was withdrawn for analysis, including biochemical, cytological, ink staining, acid-fast staining, Gram staining, bacterial culture, and pathological examination. The patient reported relief from the headache following the lumbar puncture but later experienced intermittent headaches without vomiting.

Further questioning revealed a history of fever approximately 2 weeks prior to admission, with a maximum temperature of 38.5°C. The fever was treated symptomatically at a local hospital, after which the patient’s body temperature returned to normal, and no further febrile episodes occurred.

**Figure 3.**
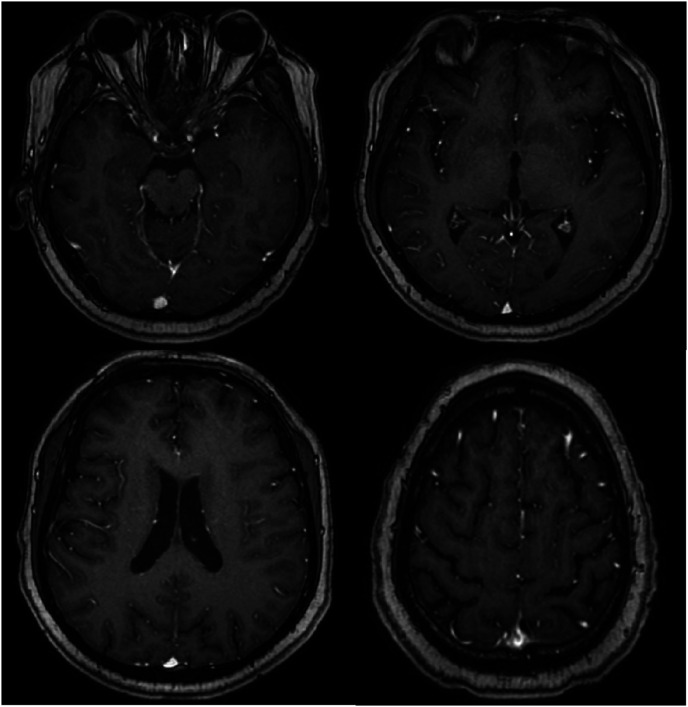
Magnetic resonance imaging of the brain, T1-weighted sequence, showing no significant abnormalities.

**Figure 4.**
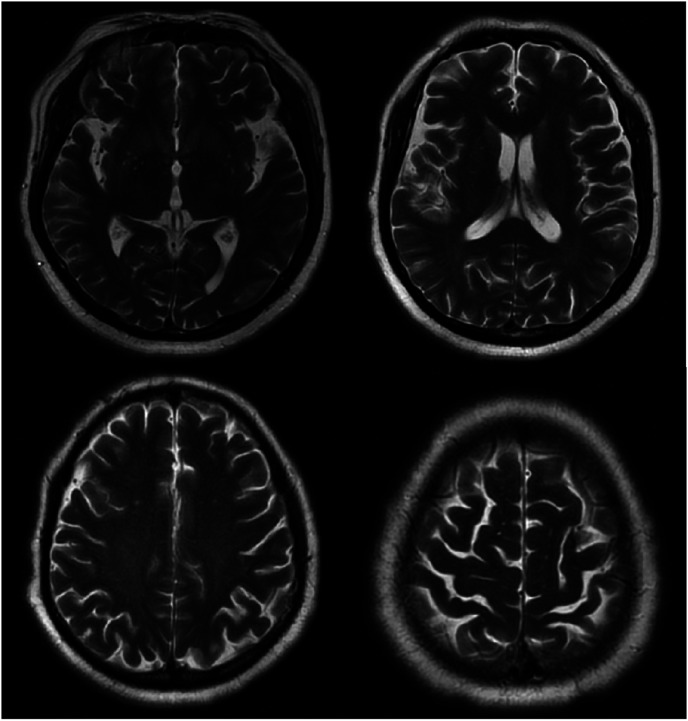
Magnetic resonance imaging of the brain, T2-weighted sequence, showing no significant abnormalities.

**Figure 5.**
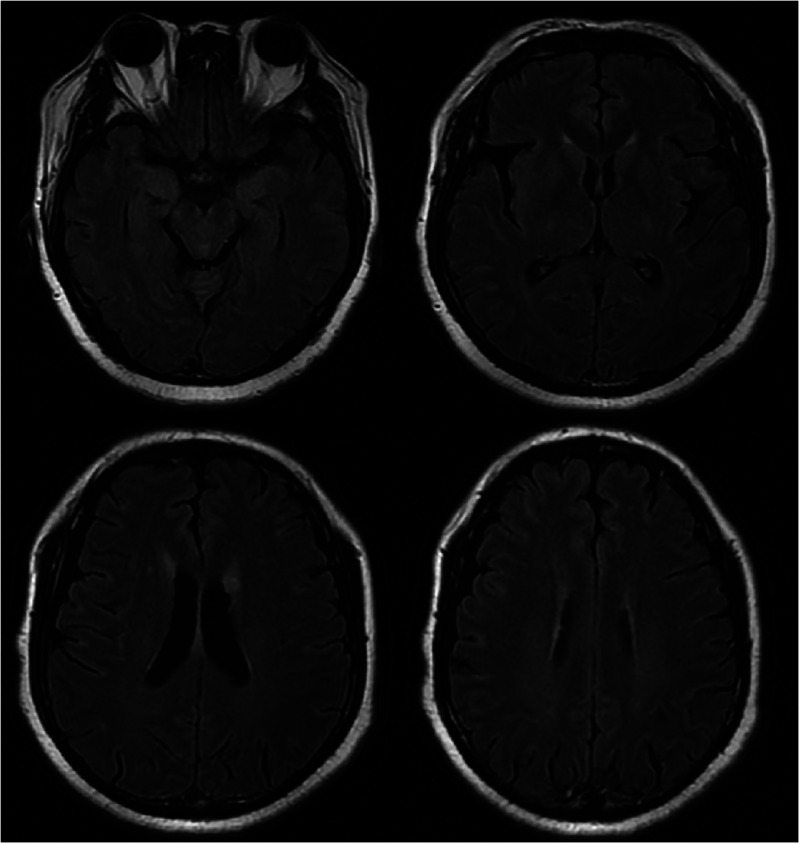
Magnetic resonance imaging of the brain, T2 fluid-attenuated inversion recovery sequence, showing no significant abnormalities.

**Figure 6.**
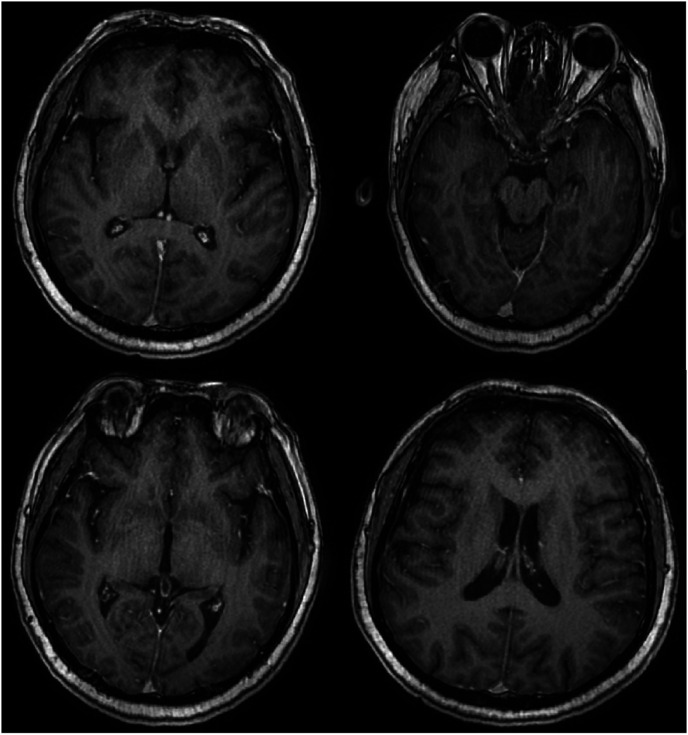
Magnetic resonance imaging of the brain using the CUBE sequence, showing no abnormal enhancement foci.

**Figure 7.**
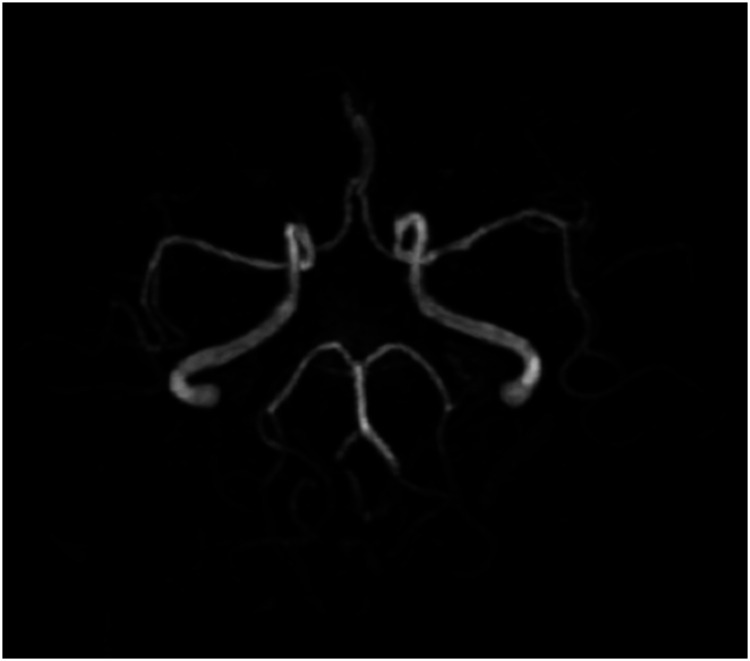
Magnetic resonance angiography of the brain showing atherosclerotic changes.

**Figure 8.**
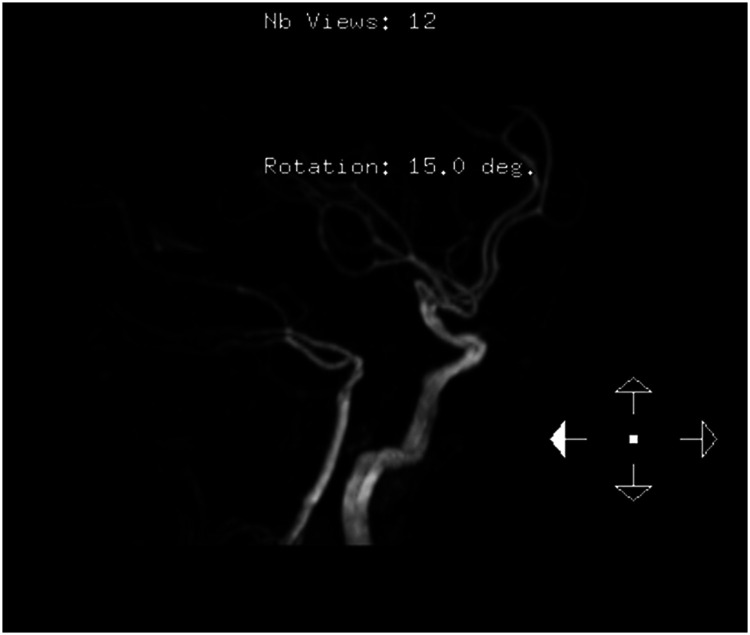
Magnetic resonance angiography of the brain showing localized stenosis in the ocular segment of the left internal carotid artery.

**Figure 9.**
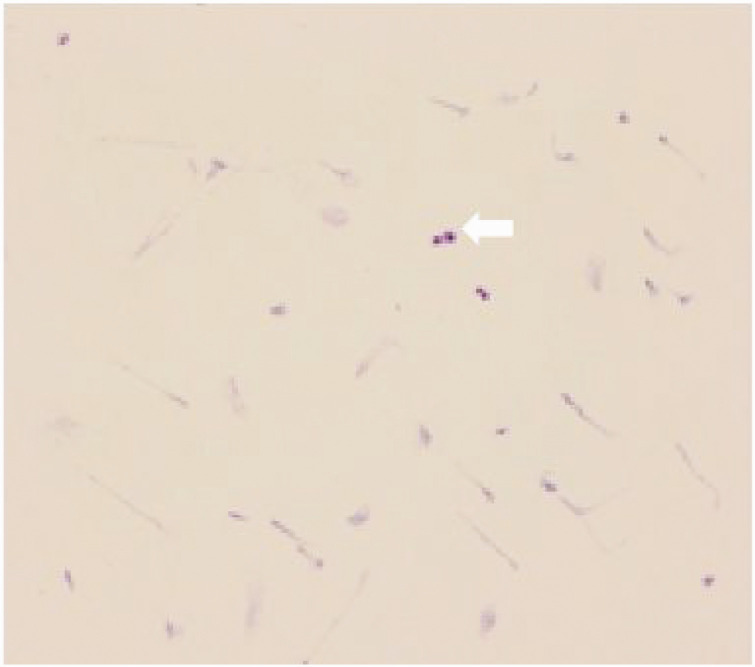
Magnetic resonance venography of the brain showing thinning of the left transverse and sigmoid sinuses compared with the contralateral side.

**Figure 10.**
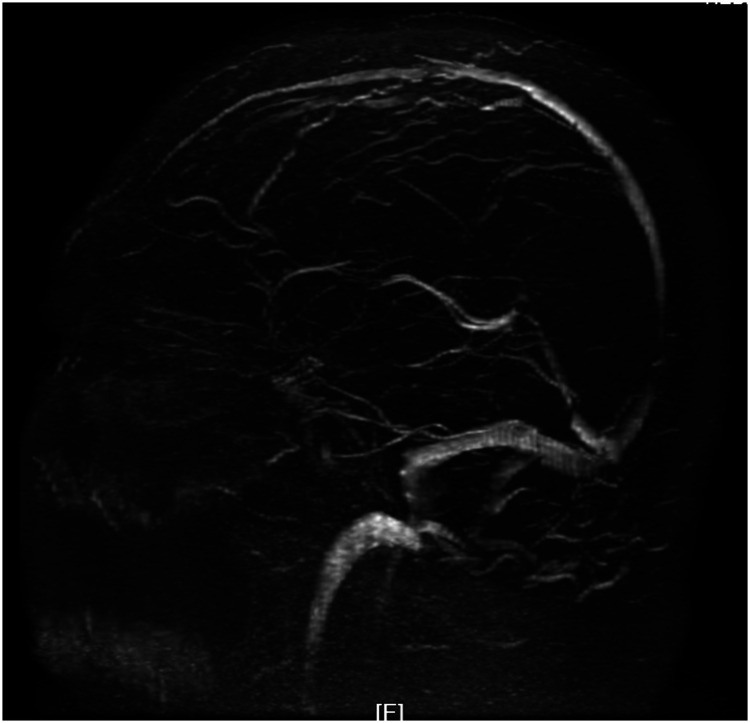
Magnetic resonance venography of the brain showing thinning of the left transverse and sigmoid sinuses compared with the contralateral side.

Magnetic resonance imaging of the brain revealed chronic small ischemic foci in the frontal lobes and an empty sella but showed no significant abnormalities (Figures 3–6). Magnetic resonance angiography and venography indicated atherosclerotic changes, with localized stenosis in the ocular segment of the left internal carotid artery (Figures 7–10). The left transverse sinus and sigmoid sinus appeared thinner than their contralateral counterparts, which was noted for clinical correlation. Diffusion-weighted imaging and susceptibility-weighted imaging showed no abnormal hyperintensities.

**Figure 11.**
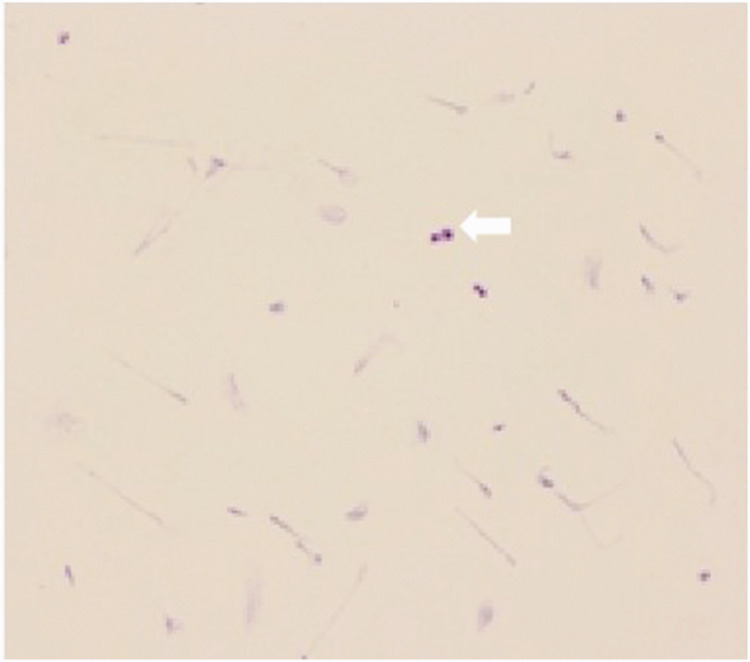
Ink staining of cerebrospinal fluid showing the presence of *Cryptococcus.*

Routine cerebrospinal fluid analysis revealed a weakly positive Paneth reaction and a leukocyte count of 83 × 10^6^/L. Biochemical examination showed a chloride concentration of 117 mmol/L, total protein of 81.72 mg/dL, and glucose of 27.48 mg/dL. Gram staining did not detect bacteria or fungi, but ink staining suggested the presence of *Cryptococcus* (Figure 11). Cytological analysis identified abnormal findings, including mixed cell populations with an increased proportion of eosinophils and basophils, 2% neutrophils, and 3% activated lymphocytes, with *Cryptococcus* observed in clusters or scattered throughout the sample. Tuberculosis-infected T-cell testing yielded results of 38 SP0s/2.5 × 10^5^ peripheral blood mononuclear cells for experiment A and 97 SFCs/2.5 × 10^5^ peripheral blood mononuclear cells for experiment B. Next-generation sequencing of the cerebrospinal fluid did not detect the *Cryptococcus neoformans* complex. Cranial meningeal CUBE imaging showed no abnormal enhancement foci.

The patient was treated with an initial dose of 800 mg of fluconazole, followed by 400 mg administered as an intravenous infusion once daily. Amphotericin B was administered intrathecally, starting with a dose of 0.2 mg on the first day, 0.4 mg on the third day, 0.6 mg on the sixth day, and 1 mg on the ninth day, with subsequent doses of 1 mg given twice weekly.

Given the high suspicion of tuberculosis, the patient and his family were fully informed about his condition, and he was transferred to an infectious disease hospital for specialized treatment. Unfortunately, at a follow-up 2 days later, it was reported that the patient had died at the infectious disease hospital.

## Discussion

Idiopathic eosinophilia is an unexplained form of eosinophilia that requires exclusion of secondary causes. Causes of secondary eosinophilia include infections, collagen–vascular disease, drug reactions, pulmonary eosinophilia, and metabolic conditions such as adrenal insufficiency.^7–10^ Nonmyeloid malignancies may also be associated with secondary eosinophilia, including T-cell lymphoma, Hodgkin lymphoma, and acute lymphoblastic leukemia.^11–13^ Rare disorders associated with eosinophilia include familial eosinophilia, hyper-IgE syndrome, Omenn syndrome, episporadic angioedema with eosinophilia (Gleich syndrome), and eosinophilia–myalgia syndrome.^14–16^ A detailed travel history and repeated ova and parasite testing, stool cultures, and antibody testing for specific parasites (e.g., *Strongyloides*) are essential to determine the etiology of infection in an appropriate clinical setting.^17^

Early case studies have demonstrated that patients with HES are at significant risk of life-threatening cardiac complications. In a retrospective study of 57 patients with HES published in 1973, congestive heart failure identified at autopsy accounted for 65% of deaths.^3^ In addition to cardiac disease, a peripheral hematopoietic or white blood cell count exceeding 100 × 10^9^/L is considered a poor prognostic factor.^18^ Other factors associated with a worse prognosis include concurrent myeloproliferative syndrome, corticosteroid-refractory hypereosinophilia, heart disease, male sex, and high eosinophil counts.^19^

Corticosteroids are the primary treatment for patients with strictly defined HES in which all other possible causes of hypereosinophilia have been excluded. Corticosteroids are highly effective and rapidly reduce eosinophil counts.^17^ Additionally, hydroxyurea and interferon-α have been shown to be effective therapies.^20–30^ Other second- and third-line agents include vincristine,^31–33^ cyclophosphamide,^33^ and etoposide.^34,35^ The use of 2-chlorodeoxyadenosine, either alone^36^ or in combination with cytarabine^37^ and cyclosporine-A,^38,39^ has also been reported.

In addition to drug therapy, interleukin (IL)-5-directed treatments are a current area of focus. Mepolizumab, approved by the U.S. Food and Drug Administration for the treatment of idiopathic HES, has been shown to effectively reduce the risk of disease flares and serve as a steroid-sparing therapy. Other approaches targeting IL-5, IL-5 receptors, and CD52 antibodies are still under investigation.^40^

Cryptococcal infection is a life-threatening opportunistic disease, with most cases occurring in individuals with cell-mediated immunodeficiency, including patients with AIDS, solid organ transplant recipients, and those with hematologic malignancies.^41–43^ The most common symptom of cryptococcal meningitis is headache, which may occur with or without a decreased level of consciousness. Intracranial pressure is often elevated, potentially resulting in cranial nerve palsy or seizures. Meningeal irritation, such as neck stiffness, is observed in less than 20% of cases.^44^ Without treatment, the infection can progress to seizures, confusion, reduced consciousness, and eventually coma, with a poor prognosis.^45^

Cryptococcal meningitis primarily affects individuals with compromised immune systems. However, cases have been documented in immunocompetent patients, often linked to corticosteroid use for other medical conditions.^46^ For example, a 13-year-old girl developed cryptococcal meningitis after 1 year of taking prednisolone at a dose of 4.5 mg/kg body weight daily for autoimmune hemolytic anemia.^46^ Similarly, a 14-year-old boy was readmitted with cryptococcal meningitis 8 weeks after being diagnosed with tuberculous meningitis and receiving corticosteroid therapy.^46^ In both cases, the development of cryptococcal meningitis was attributed to corticosteroid treatment because no additional risk factors were identified, and initial examinations showed no evidence of cryptococcal infection. Recent studies have further demonstrated that glucocorticoids increase the risk of infection and unfavorable outcomes in cryptococcal meningitis, even among patients who are neither HIV-positive nor recipients of organ transplants.^47^ Notably, a history of glucocorticoid use has been associated with the 1-year mortality rate in cases of cryptococcal meningitis.^47^

In the present case, the patient had been admitted to the Department of Cardiology 5 years previously for heart failure. A blood test at that time revealed an elevated eosinophil count. After cardiogenic causes were ruled out, a bone marrow aspirate was performed, including a smear, pathology examination, and hematologic tumor immunophenotyping. Tests for myeloproliferative neoplasm-related genes, such as *JAK2 V617F* and *PDGFRα*, were negative, and secondary causes such as parasitic infections, asthma, and allergies were excluded. The patient was subsequently diagnosed with idiopathic eosinophilia. He was hormone-sensitive and showed symptomatic improvement with methylprednisolone treatment during his hospitalization in the hematology department, after which he was discharged. Following this, he made repeated visits to the neurology and cardiology departments for symptoms including chest tightness, shortness of breath, dizziness, and fatigue. During these visits, he intermittently received intravenous methylprednisolone treatment in the outpatient clinic, which provided temporary symptom relief. On one occasion, he visited the neurology department for mild dizziness. Neurological examination revealed no localizing signs, and it was concluded that his hematological condition was likely responsible for the non-systemic dizziness. Cranial imaging was recommended, but he did not undergo the procedure. Four years previously, he was admitted to the emergency department with chest pain. An electrocardiogram showed extensive anterior myocardial infarction, with elevated troponin and cardiac enzyme levels. Because the time window for cardiac intervention had already passed, conservative treatment was provided. The patient also had a history of hypertension, ventricular aneurysm, ischemic nephropathy, kidney stones, and chronic prostatitis, among other conditions.

In summary, we have presented a case of idiopathic eosinophilia complicated by cryptococcal meningitis. This report highlights the potential complications associated with idiopathic eosinophilia and provides insight into the etiology and clinical course of cryptococcal meningitis.

## Data Availability

All data included in this study are available upon request from the corresponding author.
